# Time is lung: higher preservation of lung function in severe asthma patients after earlier mepolizumab treatment

**DOI:** 10.1183/23120541.00211-2024

**Published:** 2025-02-03

**Authors:** Francisco-Javier González-Barcala, Irina Bobolea, Javier Domínguez-Ortega, David Bañas-Conejero, Esteban Antelo-Cea, Eva Martínez-Moragón, Teresa Carrillo-Díaz, Marina Blanco-Aparicio, Christian Domingo

**Affiliations:** 1 Department of Pulmonology, H. Universitario Santiago de Compostela, Santiago de Compostela, Spain; 2 Translational Research in Airway Diseases Group (TRIAD), Health Research Institute of Santiago de Compostela (IDIS), Santiago de Compostela, Spain; 3 Department of Medicine, Universidade de Santiago de Compostela, Santiago de Compostela, Spain; 4 Department of Allergy, H. Universitario Clìnic de Barcelona, Barcelona, Spain; 5 Department of Allergy, H. Universitario La Paz, Madrid, Spain; 6 Institute for Health Research (IdiPAZ), Madrid, Spain; 7 Specialty Care Medical Department, GlaxoSmithKline, Madrid, Spain; 8 Department of Pulmonology, H. Universitario Doctor Peset, Valencia, Spain; 9 Facultad de Medicina, Universidad de Las Palmas de Gran Canaria, Las Palmas de Gran Canaria, Spain; 10 Department of Pulmonology, H. Universitario A Coruña, A Coruña, Spain; 11 Department of Pulmonary Medicine, Parc Taulí Hospital Universitari, Sabadell, Spain; 12 Institut d’Investigació i Innovació Parc Taulí (I3PT-CERCA), Sabadell, Spain; 13 Universitat Autònoma de Barcelona, Sabadell, Spain

## Abstract

**Introduction:**

Severe asthma involves a persistent inflammation of the airways that is associated with a greater risk of exacerbations. Exacerbations are associated with a higher lung function decline over time. The prevention of lung function decline could become a strategy for disease modification, and this could be more likely to happen in patients with an earlier therapeutic approach. Thus, this study means to analyse the effect of asthma duration in clinical outcomes such as lung function in patients from the REDES study. REDES was an observational real-world study that assessed the effectiveness and safety of mepolizumab 100 mg *s.c.* every 4 weeks for 12 months in 318 patients with severe asthma in Spain.

**Methods:**

This *post hoc* analysis evaluated how disease duration affected the study results through a stratification according to quartiles on their disease progression. Continuous analyses were also performed to assess the impact of confounder variables on forced expiratory volume in 1 s (FEV_1_) (%).

**Results:**

At baseline, patients with shorter time of disease had a significantly higher lung function than patients with longer asthma duration. At 12 months, pre-bronchodilator (BD) FEV_1_ values and the proportion of patients with ≥80% pre-BD FEV_1_ were higher according to a shorter disease persistence (Q1>Q2>Q3>Q4).

**Conclusion:**

These results support that time of disease persistence contributes to the lung function decline of patients with severe asthma, uncontrolled while on previous treatment, and that an earlier approach with mepolizumab may imply a higher preservation of their lung function.

## Shareable abstract

**The time from asthma diagnosis to mepolizumab initiation seems to contribute to lung function decline in severe asthma patients and this supports an earlier intervention with mepolizumab for a greater preservation of lung function** https://bit.ly/3SojNKG

## Introduction

Severe asthma (SA) is a chronic disease characterised by lung inflammation and airflow obstruction that remains uncontrolled despite optimised treatment with high-dose inhaled corticosteroids (ICS)**–**long-acting beta-agonists (LABA), or that requires this level of treatment to prevent it from becoming uncontrolled [1, 2]. Patients with SA are generally classified into different phenotypes depending on their underlying immune dysfunction, with the IL-5-dependent pathway accounting for >80% of times [3]. These patients are subject to persistent type 2 (T2) airway inflammation, characterised by elevated levels of eosinophils and pro-inflammatory cytokines such as interleukin (IL)-5, IL-4 or IL-13 [3]. Combinations of T2 inflammation biomarkers and clinical characteristics are predictors of asthma severity, worse disease control and future exacerbation risk [4].

Exacerbations are frequently characterised by an aggravation of the T2 inflammation that results in a compromised airflow obstruction needing systemic glucocorticoids, emergency visit or hospitalisation for its management [5]. Even after the exacerbation is resolved, patients will experience long-term consequences like loss of lung function [6]. In this regard, it has been described that there is in excess of 17 mL·year^−1^ loss of lung function as measured by forced expiratory volume in 1 s (FEV_1_) in asthmatic patients who suffer exacerbations compared to patients who do not [7], but there is already a higher annual decrease of FEV_1_ in asthmatic patients than in healthy controls: 38 mL·year^−1^ *versus* 22 mL·year^−1^ respectively [8, 9], and this is on top of the physiological age-related lung function decline, which is described to range between 20 and 46 mL·year^−1^ with a peak at 30 years and a nadir at 62 years [10].

Altogether, it seems that T2 inflammation and exacerbations lead to an excess loss of lung function in SA patients, and although the mechanisms behind it are not fully understood, the role of IL-5 in airway epithelial cells, fibroblasts and goblet cells is likely to contribute [11–13]. The ability of the airway tissue to repair and regenerate the self-perceived injuries, provoked by exacerbations or inflammatory effector cells (*e.g.* eosinophils), produces constant damage on patients’ lungs, leading to structural changes in the airways, including: disruption of the epithelial barrier integrity, subepithelial fibrosis and smooth muscle hypertrophy/hyperplasia. These alterations are thought to play a major role in lung function decline and may be the cause of airway remodelling in SA patients [14, 15].

Mepolizumab is a systemic anti-IL-5 therapy approved for patients with SA and eosinophilic phenotype, chronic rhinosinusitis with nasal polyps, eosinophilic granulomatosis with polyangiitis and hypereosinophilic syndrome [16]. The clinical effectiveness and safety of mepolizumab in SA is well established [17–23], but evidence about the effects of biologics in airway remodelling is still limited. The aim of this *post hoc* analysis of the REDES study is to determine the impact of the time of disease duration on lung function in patients with mepolizumab.

## Methods

REDES (GSK ID: 213172) was a retrospective, real-world, multicentric, observational cohort study enrolling patients with SA across 24 Spanish hospital asthma units.

The observational period included 12 months pre- and 12 months post-mepolizumab treatment (100 mg *s.c.* every 4 weeks). Eligibility criteria for the REDES study included: patients ≥18 years of age with a clinical diagnosis of severe uncontrolled asthma; patients who initiated mepolizumab ≥12 months before the date of inclusion in the study; and those who had ≥12 months of relevant medical records prior to enrolment. The primary end-point was the annual rate of clinically significant exacerbations. Secondary end-points included pre- and post-bronchodilator spirometry outcomes and other parameters related to asthma control, biomarkers and oral corticosteroid (OCS) treatment.

Details of the REDES study results, design and patient population have been published previously [22]. However, the influence of disease duration in asthma clinical outcomes has not been previously reported.

Owing to the retrospective nature of the REDES study and the data available, we defined the time of disease duration as the time from the age of asthma diagnosis to the age of mepolizumab initiation. Patients were then stratified in quartiles according to the time of asthma duration. Age of asthma diagnosis was included by every principal investigator from their patients’ clinical records to the study electronic Case Report Form.

Baseline features and other study assessments were analysed. Specifically, baseline features included sex, smoking status, comorbid nasal polyps, time from asthma diagnosis to mepolizumab initiation, atopic sensitisation and total IgE. Study assessments included blood eosinophil counts, Asthma Control Test (ACT) score, annual rate of clinically significant exacerbations, prednisone dose, patients discontinuing maintenance OCS and spirometry values. Notably, a benchmark of 0.23 L improvement in pre-bronchodilator FEV_1_ was established, as proposed by Santanello *et al*. [24], as a minimal patient-perceived improvement (MPPI), and the percentage of patients achieving the MPPI was calculated across the different subgroups.

Mean±sd was calculated for quantitative variables and percentages were used to describe proportions in dichotomous variables. Statistical significance defined as a p-value of <0.05 was calculated for the change of study assessments at 12 months in each group (intragroup), and at baseline, or 12 months between groups (intergroup). We performed t-tests for quantitative variables and Chi-square tests for dichotomous variables.

To determine the relation between the time of disease progression and lung function, a continuous correlation analysis was made. To minimise other factors’ influence, we performed a multivariate regression analysis, including as confounding independent variables: the time from diagnosis, age, body mass index, blood eosinophil levels at baseline, exacerbations pre-treatment and exacerbations during treatment. Pre-bronchodilator (BD) FEV_1_ % pre- and post-treatment, as well as Pre-BD FEV_1_% improvements were the three dependent variables assessed in three separate multivariate analyses utilising the same confounders. Only patients with data on all independent variables and the corresponding dependent variable were included.

**FIGURE 1:**
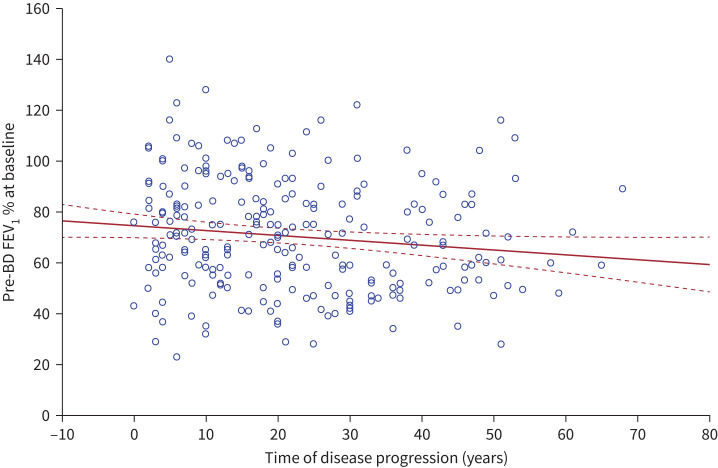
Correlation between pre-bronchodilator (BD) forced expiratory volume in 1 s (FEV_1_) % at baseline *versus* time of disease progression (years). Pre-BD FEV_1_ % – baseline = 74.547 – 0.1898 × time of disease. r= −0.1323. Solid line: correlation line; dashed lines: 95% confidence interval of the correlation analysis.

**FIGURE 2:**
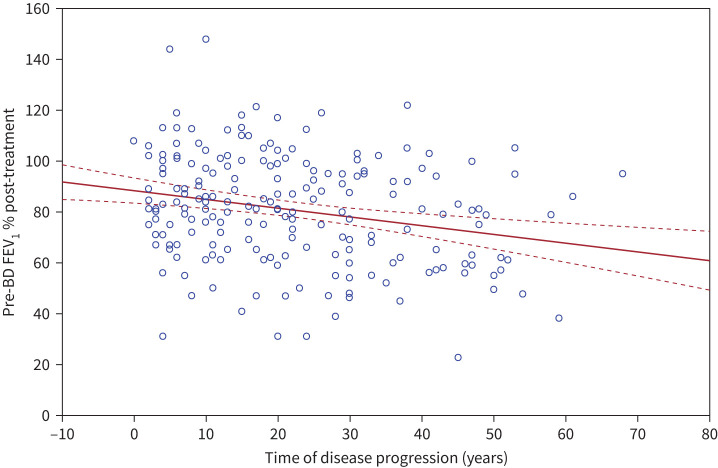
Correlation between pre-bronchodilator (BD) forced expiratory volume in 1 s (FEV_1_) % at 12 months post treatment *versus* time of disease progression (years). Pre-BD FEV_1_ % – 12 months = 88.492 – 0.3401 × time of disease. r= −0.2393. Solid line: correlation line; dashed lines: 95% confidence interval of the correlation analysis.

**FIGURE 3:**
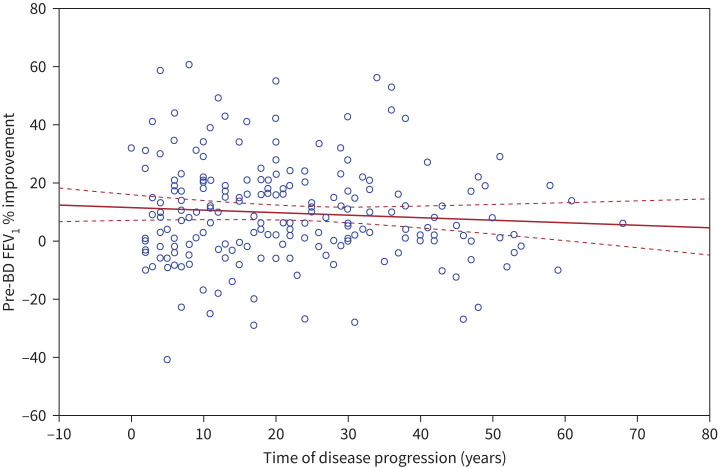
Correlation between improvement in pre-bronchodilator (BD) forced expiratory volume in 1 s (FEV_1_) % at 12 months post treatment *versus* time of disease progression (years). Pre-BD FEV_1_ % improvement = 11.563 – 0.0832 × time of disease. r= −0.0715. Solid line: correlation line; dashed lines: 95% confidence interval of the correlation analysis.

Three graphs were then created using bivariate models to assess the correlation between time of disease progression and: 1) pre-BD FEV_1_ % at baseline; 2) pre-BD FEV_1_ % after 12 months of treatment; and 3) improvements in FEV_1_ % (figures 1, 2 and 3).

These analyses were performed using the program STATISTICA v.13.5.0.63.

## Results

From the total 318 patients of the REDES study, the age of asthma diagnosis had not been collected in 17; therefore, they were excluded from this analysis. 84 patients entered in Q1 (≤10 years), 74 patients in Q2 (11–20 years), 70 patients in Q3 (21–33 years) and 73 patients in Q4 (≥34 years) (tables 1 and 2).

**TABLE 1:** Baseline features based on quartiles stratification

Baseline features	N	Q1	N	Q2	N	Q3	N	Q4
**Sex, female, n (%)**	84	57 (68)	74	51 (69)	70	50 (71)	73	52 (71)
**Comorbid nasal polyps, n (%)**	84	39 (46)	74	29 (39)	70	39 (56)	73	35 (48)
**Age years, mean±sd**	84	53.4±13.8	74	58.7±13.5	70	53.5±12	73	61.1±8.8
**Age at asthma diagnosis years, mean±sd**	84	47.7±14.3	74	42.9±13.4	70	26.5±3.8	73	16.5±9.9
**Years from diagnosis to mepolizumab, mean±sd**	84	5.7±2.8	74	15.8±3.0	70	26.8±3.8	73	44.6±7.7
**Nonsmokers, n/N (%)**	80	46/80 (57.5)	71	51/71 (71.83)	69	46/69 (66.67)	70	47/70 (67.14)
**Atopic sensitisation, n (%)**	82	40 (49)	74	30 (40.5)	70	25 (35.7)	73	28 (38.4)
**Total IgE IU, mean±sd**	83	340.6±456.4	67	363.6±520.8	69	348.2±618.5	69	310.5±506.1

**TABLE 2:** Study assessments based on quartiles stratification

Study assessments	Q1				Q2				Q3				Q4			
	N	B	N	12M	N	B	N	12M	N	B	N	12M	N	B	N	12M
**Blood** **eosinophil counts (cells·µL^−1^), mean±sd**	84	829.88±1051.35	58	**113.10**±**214.73**	73	619.47±446.96	54	**136.33**±**417.44**	70	810.57±1204.46	53	**70.09±38.33**	73	629.19±370.17	51	**80.41±84.10**
**ACT score, mean±sd**	75	12.95±4.44)*^,+,#^	69	**21.03**±**4.10**	64	14.66±5.52*	59	**21.46**±**3.13**	64	14.47±5.08^+^	62	**20.48±3.96**	70	14.43±5.03^#^	61	**20.59±3.95**
**Patients with ACT score ≥20, n/N (%)**	75	5/75 (6.67)*	69	**55/69 (79.71)**	64	15/64 (23.44)*	59	**44/59 (74.58)**	64	10/64 (15.63)	62	**44/62 (70.97)**	70	11/70 (15.71)	61	**43/61 (70.49)**
**Annual exacerbations, mean±sd**	84	4.58±3.32	84	**1.05**±**1.44**	74	3.97±3.09*	74	**0.80**±**1.19**	70	5.21±4.18*	70	**1.20±1.71**	73	4.34±3.50	73	**0.89±1.20**
**Prednisone dose mg·day^−1^, mean±sd**	36	11.25±9.07	34	**3.31**±**4.65**	32	11.43±9.50	32	**4.48**±**6.57**	26	13.17±11.31	22	**5.11±7.89**	23	9.53±7.42	25	**3.23±6.25**
**Patients with maintenance prednisone at baseline and 0 mg·day^−1^ at 12 months, n/N (%)**	28		28	16/28 (57.14)	25		25	9/25 (36)	16		16	8/16 (50)	16		16	9/16 (56.25)

Data in bold indicates p<0.05 for the difference at 12 months *versus* baseline. B: baseline; 12M: 12 months post treatment; ACT: Asthma Control Test.

*^,+,#^: p<0.05 for intergroup difference at baseline.

Mean age was 53.4, 58.7, 53.5 and 61.1 years and mean time from asthma diagnosis until mepolizumab initiation was 5.7, 15.8, 26.8 and 44.6 years in Q1, Q2, Q3 and Q4, respectively. Other baseline characteristics for patients are shown in tables 1 and 2.

A consistent reduction in annual exacerbations was seen across all groups. Regarding asthma symptoms, ACT also improved across quartiles subgroups without an apparent influence of the time of disease persistence. OCS dose and the proportion of patients requiring maintenance OCS decreased similarly (tables 1 and 2).

Regarding lung function, at baseline, Q1 had a statistically significant higher mean pre-BD FEV_1_ % predicted of 74.7% compared to 67.2% in Q3 and 66.3% in Q4, and numerically higher compared to Q2 (71.7%) (table 3).

**TABLE 3:** Spirometry values based on quartiles stratification

Spirometry values	Q1				Q2				Q3				Q4			
	N	B	N	12M	N	B	N	12M	N	B	N	12M	N	B	N	12M
**Pre-bronchodilator FEV_1_ L, mean±sd**	73	2.09±0.78*^,+^	59	**2.37**±**0.72^¥,§^**	61	1.85±0.71*^,#^	51	2.04±0.73^¥,¶^	55	1.92±0.84^ø^	49	**2.16±0.86^ƀ^**	55	1.61±0.61^+,#,ø^	43	1.67±0.48^§,¶,ƀ^
**Pre-bronchodilator FEV_1_ %, mean±sd**	74	74.73±24.01*^,+^	59	**87.03**±**20.68^¥,§^**	61	71.68±20.44	52	**84.13**±**20.87**	56	67.24±22.74*	49	**77.03±21.14^¥^**	55	66.35±19.71^+^	43	**73.74±21.42^§^**
**Patients with pre-BD FEV_1_ % improvement >0.230** **L, n/N (%)**	57		57	27/57 (47.37)	51		51	24/51 (47.06)	47		47	18/47 (38.3)	41		41	12/41 (29.27)
**Patients with pre-BD FEV_1_ % ≥80%, n/N (%)**	74	30/74 (40.54)	59	**40/59 (67.80)^¥,§^**	61	21/61 (34.43)	52	**32/52 (61.54*)***	56	18/56 (32.14)	49	23/49 (46.94)^¥^	55	16/55 (29.09)	43	18/43 (41.86)^§^
**Post-bronchodilator FEV_1_ L, mean±sd**	54	2.24±0.79*	30	2.37±0.69^¥,§^	53	2.02±0.66^+^	39	2.09±0.53^¥,¶^	48	2.04±0.75^ø^	37	2.29±0.72^ƀ^	47	1.77±0.69*^,+^	42	1.83±0.70^§,¶,ƀ^
**Post-bronchodilator FEV_1_ %, mean±sd**	54	80.63±24.90*	33	87.73±24.29^¥^	51	79.40±21.05^+^	40	85.57±22.22^§^	48	73.93±22.53	37	**85.18±18.98^¶^**	47	71.73±23.34*^,+^	44	76.93±22.81^¥,§,¶^
**Patients with post-BD FEV_1_ % ≥80%, n/N (%)**	54	30/54 (55.56)	33	23/33 (69.70)	51	25/51 (49.02)	40	26/40 (65.00)	48	21/48 (43.75)	37	22/37 (59.46)	47	18/47 (38.30)	44	21/44 (47.73)
**Pre-bronchodilator FVC %, mean±sd**	74	90.50±21.03	59	**97.36±19.60**	61	88.86±21.63	52	**97.53±22.40**	56	89.29±17.68	49	**97.70±16.60**	54	85.84±17.66	43	**93.43±22.38**
**Post-bronchodilator FVC %, mean±sd**	53	89.13±23.81	32	97.59±20.84	50	91.34±23.97	38	94.74±24.74	48	93.14±23.02	38	100.89±22.52	47	89.44±23.96	44	97.74±24.59

Data in bold indicates p<0.05 for the difference at 12 months *versus* baseline. B: baseline; 12M: 12 months post treatment; FEV_1_: forced expiratory volume in 1 s; BD: bronchodilator; FVC: forced vital capacity. *^,+,#,ø^: p<0.05 for intergroup difference at baseline. ^¥,§,¶,ƀ^: p<0.05 for intergroup difference at 12 months.

**FIGURE 4:**
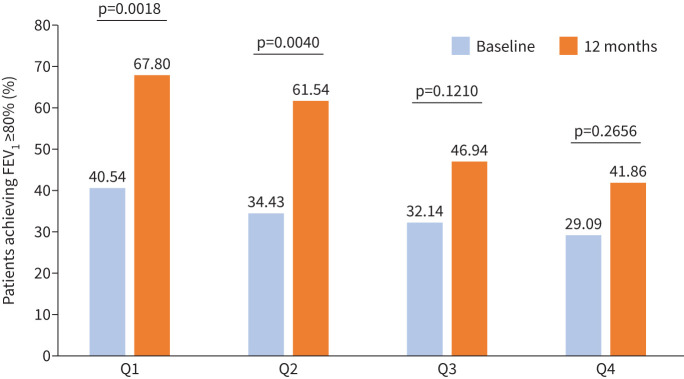
Proportion of patients achieving forced expiratory volume in 1 s (FEV_1_) normalisation (≥80%) in the quartiles stratification.

**FIGURE 5:**
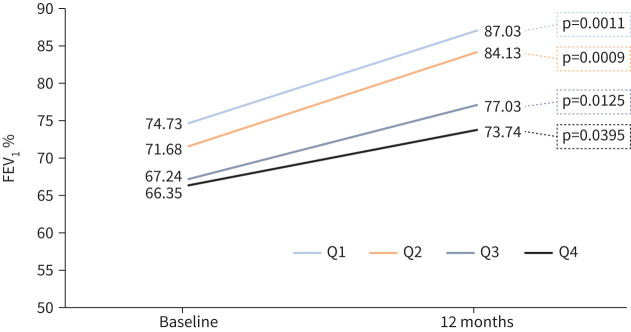
Mean change of % forced expiratory volume in 1 s (FEV_1_) after 12 months of mepolizumab treatment in the quartiles stratification.

Figures 4 and 5 illustrate that both pre-BD FEV_1_% value and the proportion of patients with ≥80% pre-BD FEV_1_ were higher as the disease duration was shorter (Q1>Q2>Q3>Q4); this was observed both at baseline and at 12 months (table 3). Also, a downward trend was shown in pre-BD FEV_1_ % improvement after treatment: 12.30, 12.45, 9.79 and 7.39 (%), respectively. Comparable results were seen in post-BD FEV_1_ %, although with more variability than pre-BD (table 3).

Looking at the proportion of patients who achieved the MPPI of 0.23 L, no statistically significant differences were observed between quartiles, although it numerically decreased from Q1 to Q4 (table 3).

Regarding the multivariate regression, at baseline, only the time of disease progression has a statistically significant negative correlation with lung function preservation measured with pre-BD FEV_1_ % (b*= −0.171906, p=0.020169). While at 12 months, both the time of disease progression and the number of exacerbations during the 12-month treatment period showed statistically significant negative correlation with pre-BD FEV1 % (b*= −0.232806, p=0.002332; b*= −0.205309, p=0.008932, respectively).

No statistically significant associations were found when measuring the improvement in pre-BD FEV1 % pre- and post-treatment (b*= −0.065667, p=0.373923).

Bivariate correlation between pre-BD FEV_1_ %, pre- and post-treatment, and time of disease progression are illustrated in figures 1 and 2.

## Discussion

It is well established that an early intervention in some inflammatory diseases, with proper pharmacological agents, can halt the biological processes associated with the disease and prevent disease progression [25].

In SA this concept is not that extended yet. However, eosinophilic functions and related damage within the airways influence the remodelling process of diseases such as SA and chronic rhinosinusitis with nasal polyps [14]. Moreover, it has been reported that targeting IL-5 with mepolizumab significantly reduces the expression of airway remodelling parameters like the accumulation of tenascin, lumican and procollagen III in the bronchial mucosal reticular basement membrane [26].

In our study, patients with a prompter introduction of mepolizumab since their asthma diagnosis showed a more preserved lung function as measured by FEV_1_ pre- and post-BD. Moreover, these patients achieved higher values of lung function at 12 months, compared to patients who had a later mepolizumab initiation.

Consistent with these findings, multivariate regression analyses showed an influence of the time of disease progression on the preservation of lung function at baseline, and on achieving higher values after 12 months of treatment with mepolizumab, being the latest also influenced by the patients’ exacerbations during treatment.

These results support that the time of disease duration contributes to the decline of lung capacity, and that an earlier therapeutic approach with mepolizumab could imply a higher preservation of lung function. These are also aligned with previously published evidence about clinical remission, where patients that achieved clinical remission showed a less severe form of SA compared to those who did not [27]. Thus, the moment of mepolizumab initiation is crucial in achieving the best possible outcomes, as the precise targeting of T2 inflammation with mepolizumab (anti-IL-5) could interrupt airway remodelling.

Owing to its retrospective nature, this study has several limitations. The age at which ICS treatment began was not documented, so it could not be ensured that patients were treated with ICS after their asthma diagnosis in a timely manner. No availability of quality-of-life questionnaires was another important factor, although it has been reported that lung function improvement does not necessarily correlate with a better quality of life [28].

The relevance of lung function has not been put in question in asthma. It is, in fact, one of the main variables to define clinical remission, and several studies point out that a better-preserved lung function at baseline increased the odds of achieving clinical remission in severely asthmatic patients, after biologic treatment [29, 30]. This reinforces the existence of an association between lung function and the course of the disease.

Apart from this, tobacco usage is a major confounding factor when measuring lung function; in this regard, the smoking status of the four groups was balanced. It should be noted that there were just three current smokers in REDES, all three of them being included in the first quartile, and the rest being “ex-smokers” or “non-smokers” in this study.

### Conclusion

The results of this study suggest that the duration of disease contributes to lung function decline of SA patients, uncontrolled on previous treatment, and support an earlier intervention with mepolizumab for a higher preservation of lung function.
